# 

**Published:** 1922-01

**Authors:** 


### HOSPITAL and HEALTH REVIEW

*Established 1886* *No.* 1846. *Vol. LXXI.*

*New Series.* Vol. 1. No. 4 *January* 1922 Price Sixpence

CONTENTS.

| | PAGE |
|---|---|
| Notes and Comments . . . . . . . . | 93 |
| Prevention and Cure . . . . . . . S. H. Daukes, O.B.E., M.B., D.P.H., D.T.M. & H. | 97 |
| The Renewal of Vision in Hospital Administration. Alexander Pym. | 99 |
| Notable Epidemics. III.—The Great Plague A. T. Nankivell, M.D., D.P.H. | 101 |
| Review of the Month . . . . . . . . | 103 |
| The Government and the Voluntary System | 106 |
| The Treatment of Chronic Otorrhœa in School Children . . . . . A. R. Friel, M.D., F.R.C.S.I. | 107 |
| A New Year's Appeal . . . . . . . . | 109 |
| The Period of Infectivity of Venereal Disease. | 112 |
| The Control of Diphtheria . . . . . . | 113 |
| Passing Events and Topics . . . . . . | 115 |
| Reviews of Books. . . . . . . . . | 117 |
| Our Leaflet Competition: Correspondence. | 120 |

